# Dental Law and Dental Education

**Published:** 1892-11

**Authors:** W. E. Magill

**Affiliations:** Erie, Pa.


### International Dental Journal.

Vol. XIII. November, 1892. No. 11.

### Original Communications.[1]

### DENTAL LAW AND DENTAL EDUCATION.[2]

BY W. E. MAGILL, D.D.S., ERIE, PA.

As members of our profession we are all interested in dental law and dental education: in law, as a check upon vice, a restraint put upon charlatanism; in education, as a beneficent, uplifting force, urging towards higher standards and better attainments.

Law is the result of discovered needs, the evidence of a desire to change the existing order of things. Especially is this true of dental law. For many years we were contented and comfortable without legislation, unconscious of any benefits to be derived from that direction, and it may be interesting to inquire whence came the innovating impulse to disturb this placidity of professional life. I think it may be traced to the influence of college education. Conscious of the benefits derived from enlarged opportunities, generous men desired to elevate the profession by establishing higher standards. Some, no doubt, labored for the public good, desiring to protect the community, so far as is possible, by legislation against the evils of ignorance and quackery.

[1] The editor and publishers are not responsible for the views of authors of papers published in this department, nor for any claim to novelty, or otherwise, that may be made by them. No papers will be received for this department that have appeared in any other journal published in the country.

[2] Abstract of paper read at Union Meeting of Pennsylvania and New Jersey Societies, July 20, 21, 22, 1892.

It may be true elsewhere, and I apprehend it is true in every State, as it is here in Pennsylvania, that all movements in favor of dental legislation have originated within the profession. While the ultimate and important effect of legislation is to guard well the interests of the people, outside of the profession, not from them has come the demand for laws to regulate practice, with a view to improved and more efficient professional knowledge. Indeed, there has been a disposition to gather, with local pride, around the uneducated and defend them, as if any law to compel education was an interference with inherent rights of "life, liberty, and the pursuit of happiness."

But the most active opposition to the enactment of restraining law has come from conservatism within our profession, from men established in practice under the old order of affairs, and who feared or disliked innovation, lest in some way it might unfavorably affect themselves. To disarm and conciliate this adverse influence, in all the earlier laws it was necessary to demand less and be satisfied with less than was desirable, on the part of those who asked for legislation, and who obtained what they could, trusting to the future for better laws, when experience should have proved the benefit to be expected from them. Bearing this in mind, you will be lenient in criticism of those laws first enacted, for they were the first steps cut in the face of a high rock of prejudice, beyond which our professional life has expanded, and now we stand with a wider horizon and more glorious prospect, union in our ranks, and professional jealousy banished, we hope, forever.

One of the beneficent effects of law in a State is to compel legislation in adjoining States. In Pennsylvania we were not fully conscious of the value of law until this became the dumping-ground for those incapables who were by legal enactments thrown out of other States.

In July, 1868, the Susquehanna Dental Association, then in session at Scranton, passed the following resolution:

"*Resolved,* That in view of the legislation obtained in several other States, this Association will most heartily co-operate with other dental Associations in any effort that may be made with a view to securing legislative action which shall define the qualifications of practitioners of dentistry in this Commonwealth."

In November of that year a call was published in the *Dental Cosmos* for a State Dental Convention, to be held in Philadelphia, December 8, 1868, "for the purpose of forming a State Dental Society." This organization was urged with the express view to secure "legislative action to regulate the practice of dentistry within the State." It is thus shown that the very foundation of our associated life as a State Society is the desire to have clearly defined by statute what should properly be required, in the way of preparation and acquirement, of the men who proposed to enter this respectable profession, which had proved its value and importance to the health and comfort of the people.

Eight years of effort were required to secure the desired enactment, which became a law in April, 1876. The Pennsylvania State Dental Society, true to the object of its organization, was earnest in effort to secure legislation, and has been diligent to obtain enforcement of the law, which is one of the simplest and least elaborate to be found in the statute books of the States. It is not perfect. It is lacking in definition of what shall constitute practice. It does not assign to any public officer the duty of prosecuting offenders, nor does it provide a fund to meet the necessary expense to be incurred in enforcement. Under its provisions satisfactory work has only been done where a local Society, operating within a given district, has raised money and energetically undertaken the prosecution of those who were in unlawful practice. Notably in Pittsburg the value and soundness of the law have been proven, and in other portions of the State a few decisions by judges have so sustained the law that now no doubt exists of our ability to enforce its provisions, provided we have sufficient testimony to offer and money to pay expenses.

An amendment, secured in 1883, requires the registration of all dentists, either by diploma, certificate of State Examining Board, or affidavit setting forth time and place of continuous practice.

The public sentiment which sustains a law is of more value than the law, for without the support of such sentiment the law is worthless. Serious difficulty was found, in early attempts to enforce the law, on account of existing prejudice on the part of judge and jury, who considered its requirements arbitrary and unjust.

I cannot speak with authority of the effect of legislation upon the profession in other States, but here the influence in the direction of education has been distinct and beneficial. Men who had already commenced practice, with no preparation but a brief pupilage, were induced to attend college and graduate. Young men came before the Examining Board, and, failing to pass examination, returned home to study and prepare for college. The number of those who have applied to the board for certificates has decreased year by year, until now the apparent understanding is, that the man who desires to enter practice must attend college. This is due to steady advance in the requirements of the Examining Board, and to progressive opinion in and out of the profession.

In these days we hear and read much in regard to dental law and dental education. There is an evident feeling of unrest, an agitation for something to be desired but not yet attained. And yet nearly every State has its law, and, we might almost say, its college. Certainly there never was a time when opportunities were so numerous to obtain what is called education in institutions proposing to teach students in dentistry.

In certain places much fault is found with the result of college education, and the demand is for fewer schools, more thoroughness in methods, and less strife for the longest list of matriculants. While admitting that there may be good grounds for such a demand, to me it seems more important that the character of matriculates and graduates should be carefully considered. As a rule, the honest man will be faithful in what he does to the extent of his ability, and the inevitable result of such faithfulness is improvement, advancement. The dishonest practitioner is an uncertain quantity, whatever his attainments, and with him careful operations depend upon mere whim or fancy for a patient, or opportunity to deceive. I do not feel much inclined to find fault with the man who thinks he is too poor to attend college, but obtains all the knowledge within his reach, and faithfully does his best for his patients; but I am disposed to censure severely the man who receives a diploma from a respectable college, and then trails it in the mire of disreputable methods, by which he expects to draw to himself business which, in the proper order of things, should go to another and better man. These so-called educated men, without correct principles, are the bane of professional life and character today. Having complied with the letter of the law and obtained a diploma, the man of this stamp is safe, as regards the law, in his disreputable methods, and taints the professional atmosphere with an odor which no disinfectant yet discovered can neutralize. Of course the modest man, who has high regard for the character and reputation of his profession, is at a disadvantage when these men who adopt the *rôle* of charlatan come between him and the public, waving their banners bearing cuts of artificial teeth, hanging out the immense gilded imitation of an upper first molar, and reciting tales of wonderful exploits in remedying defects real or imaginary. Unfortunately for the good man, a large portion of the community upon which he depends for support consists of those who judge superficially, or are easily deceived by large pretensions, or have had small experience in the world. While he has a following that is satisfactory in quality, it may not be so in quantity. The truth is, that one of our serious wants, as a profession, is education among the people, who can demand and obtain what they need, whenever competent to judge, but will continue to be imposed upon so long as they lack knowledge of what constitutes professional ability and shrewdness in comprehending character.

Possibly we should demand and expect the college to stand between the public and the unworthy student; but, to do this effectually, in many cases the college must have the right to revoke or recall its diploma. We may not yet be ready for this advance in legislation, but the necessity for some such safeguard is already admitted, and the evil being known, we may safely depend upon the wisdom of our profession and popular enlightenment yet to come to work a reform.

We readily concede to the college the paramount influence in dental education to-day, but those whose professional experience dates back many years can remember a time when as an educational force the Association was greater than the college. Indeed, it may be questioned if even now laurels should not be divided. The direct influence of the college is limited to three years, whereas the Association continues to shed light and influence during all the years of one's professional life. It is a school where experience teaches and discussion draws out truth, and many opinions converge upon a given subject.

As a Society we have not undertaken the task of education, but by giving active support to the law we have contributed largely to the success of colleges, and thus to education. In return, we are indebted to these institutions for the cultivated men their ranks have furnished and for valuable contributions in all departments of professional knowledge. To the colleges we have a right to look for all that is valuable in progress and grand in principle. Our disposition is not to repress or discourage those who are doing a good work, as some surely are. We would rather unfurl a banner inscribed "Excelsior!" and, pointing to the legend, bid them God-speed in the upward way.

As to legislation which shall require in every State the examination, by boards appointed for the purpose, of all who propose to begin the practice of dentistry, we see a drift of sentiment in that direction, but hope for such prompt and thorough action on the part of colleges as shall forestall the movement and render it unnecessary. It is a wretched stigma to attach to our American institutions that their diplomas lie, and are of no value as evidence of preparation for practice. There is no necessity for any college to continue long under such a ban, and each should strive to be the first to prove to the profession and the world its determination to be free hereafter from even a suspicion of such incompetency as is charged.

As pertinent to the subject let me quote from a recent article on preparatory schools: "What, under the existing state of things, may be justly demanded? It is evident that there are many things, in themselves eminently desirable, that must not be expected, nor even asked for. The school cannot create brains. A certain per cent. of students never ought to attempt preparation for college, and are yet always to be looked for in preparatory classes so long as fond parents have their being. Even here the school can render a great service to the whole student body by shutting such incapables out of their higher classes through a system of rigid examinations, thus practically preventing them from ever reaching college halls."

This indicates a remedy within reach of every college. The lengthened term gives each institution what is equivalent to a preparatory school in its first year, and beyond that year no student should be permitted to go who has not shown himself fully up to all the standards as to character, skill, and intellect.

That there are serious defects in the present system of education pursued in some colleges we are obliged to admit. Every one who has much experience in employing young men recently graduated, is forced to the conclusion that somewhere a fault exists which should be remedied. Whence shall the remedy come? Unquestionably it should come from the college. What hinders such reform? The commercial spirit, which has too great preponderance in the origin and conduct of schools. How can this spirit be regulated or placed under control? By concentrating the influence of the profession upon this subject and in favor of reform, possibly by bringing the powers of the State to bear in exercising strict supervision of colleges and making them accountable.

We have seen that for dental laws now in existence our profession is mainly responsible, and the number is increasing rapidly. We will also be held responsible for our fostering care of colleges, to which we must look for all that is highest and best in dental education.

